# Boerhaave's Syndrome: Still a Diagnostic and Therapeutic Challenge in the 21st Century

**DOI:** 10.1155/2013/161286

**Published:** 2013-06-09

**Authors:** J. Spapen, J. De Regt, K. Nieboer, G. Verfaillie, P. M. Honoré, H. Spapen

**Affiliations:** ^1^Department of Internal Medicine, University Hospital, Vrije Universiteit Brussel, Laarbeeklaan 101, 1090 Brussels, Belgium; ^2^Department of Intensive Care, University Hospital, Vrije Universiteit Brussel, Laarbeeklaan 101, 1090 Brussels, Belgium; ^3^Department of Radiology, University Hospital, Vrije Universiteit Brussel, Laarbeeklaan 101, 1090 Brussels, Belgium; ^4^Department of Thoracic Surgery, University Hospital, Vrije Universiteit Brussel, Laarbeeklaan 101, 1090 Brussels, Belgium

## Abstract

Boerhaave's syndrome is a rare but potentially fatal condition characterised by a transmural tear of the distal oesophagus induced by a sudden increase in pressure. Diagnosis is challenging as the classic triad of vomiting, abdominal or chest pain, and subcutaneous emphysema is absent in many patients. Management is multidisciplinary and relies on rapid, distinct, and repeated imaging. Treatment has not been standardised and may be conservative, endoscopic, or surgical. We present a typical case which illustrates possible diagnostic pitfalls and the therapeutic conundrum surrounding management of the syndrome. Based on time of presentation and eventual presence of sepsis, a therapeutic algorithm is proposed.

## 1. Introduction

Boerhaave's syndrome, first described in the 18th century by the Dutch physician Herman Boerhaave, refers to an oesophageal tear caused by an abrupt rise of intraluminal pressure [1]. It is a rare but life-threatening condition that requires urgent diagnosis and treatment. If treatment is delayed, severe and potentially lethal complications such as mediastinitis, pleural empyema, septic shock, and multiple organ failure may develop. A high index of suspicion is imperative for timely diagnosis and to assure well-selected radiological and endoscopic investigations. Prompt and adequate therapy reduces mortality. Some centers advocate early and extensive surgery as the cornerstone of treatment, yet others prefer a more conservative, endoscopic, or less invasive surgical approach. Such experience-based difference in attitude likely explains the lack of well-established treatment guidelines. 

## 2. Case Report

**Figure 1:**
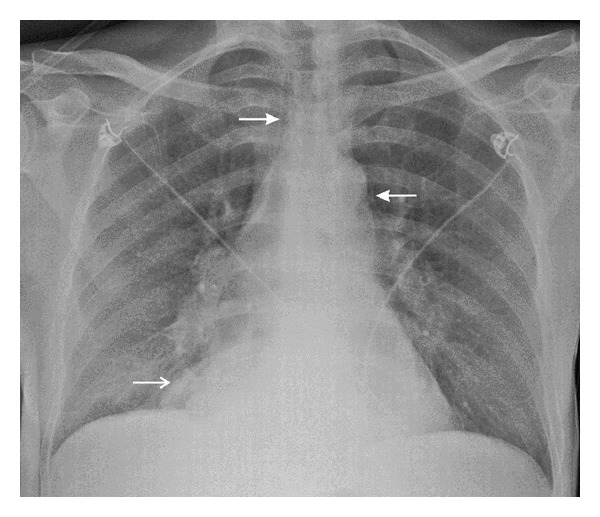
Chest X-ray at admission at the emergency ward showing a pneumomediastinum (closed arrows) and silhouette sign over the right heart border (open arrow). No pleural effusions were observed in the costolateral sinuses.

**Figure 2:**
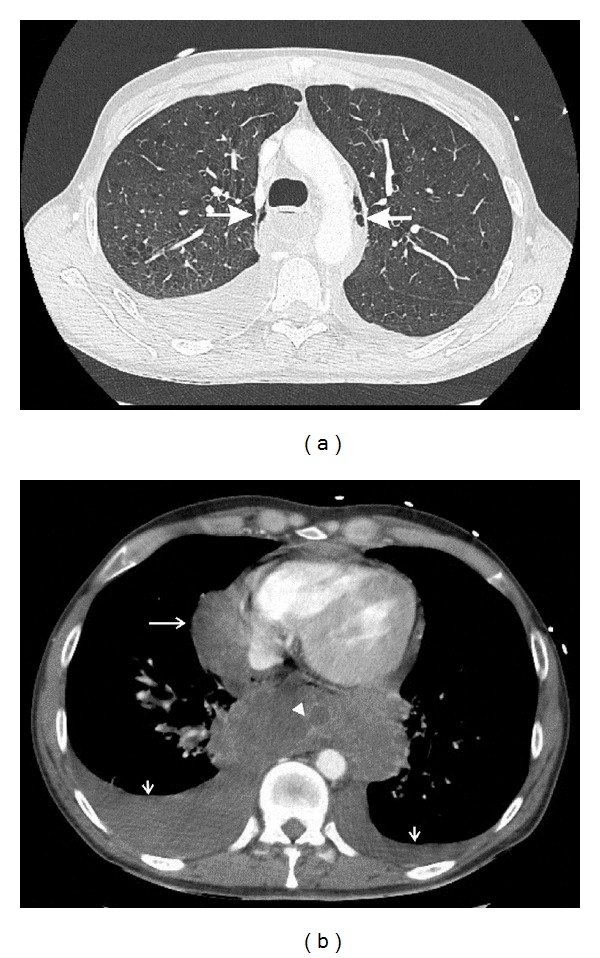
Contrast-enhanced CT scan of the thorax: (a) lung window, confirming pneumomediastinum (closed arrows), and (b) mediastinal window showing a tear in the right posterolateral wall of the distal oesophagus (arrowhead), bilateral pleural effusions (short arrows), massive retrocardiac collections, and a paracardial collection (open arrow) corresponding with the silhouette sign on chest X-ray.

**Figure 3:**
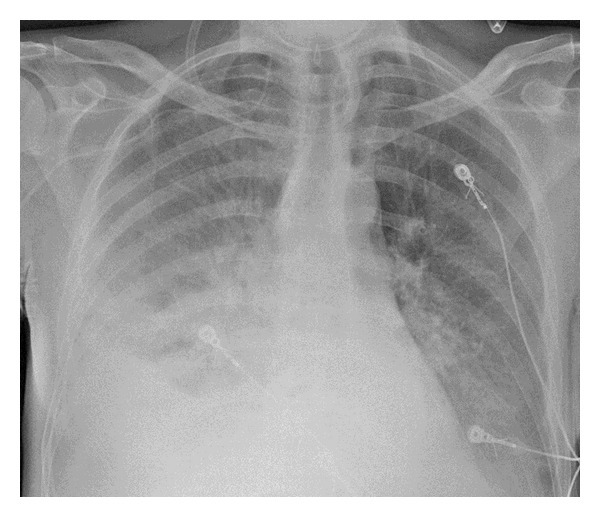
Chest X-ray, 2 h after admission, revealing a rapidly evolving right pleural effusion.

A 41-year-old man with an extensive medical history, including Child-Pugh B liver cirrhosis and chronic pancreatitis, consulted his family doctor with progressive dyspnea, retrosternal pain radiating to the back, repeated vomiting, and fever for 3 days. On admission at the emergency ward, the patient appeared confused and agitated. Core temperature was 38.5°C, heart rate was regular at 150 beats/min, and respiratory rate was 40 breaths/min. Physical examination revealed a supple but tender abdomen, normal peristalsis, and muffled breath sounds over the right lung. Blood analysis showed macrocytic anemia, 18500 leukocytes/mm³, normal enzymes, a C-reactive protein (CRP) of 303 mg/L, and a lactate level of 7.4 mmol/L. Chest X-ray showed a silhouette sign over the right heart border and small mediastinal radiolucent streaks of air (Figure 1). A contrast-enhanced computed tomography (CT) scan of the thorax confirmed the pneumomediastinum (Figure 2(a)) and showed a dilated oesophagus with a distal tear, bilateral pleural effusions, and heterogeneous retro- and paracardiac collections (Figure 2(b)). The diagnosis of Boerhaave's syndrome was made. Blood cultures were taken; intravenous antibiotic therapy (*β*-lactam + aminoglycoside) and ample fluid resuscitation were initiated. The patient was transferred to the ICU. Two hours after ICU admission, the patient became increasingly oxygen- and vasopressor-dependent. A new chest X-ray revealed a rapidly evolving right pleural effusion (Figure 3). Subsequently, the patient was intubated and mechanically ventilated. A chest tube was inserted in the right pleural space. A brown, foul-smelling fluid was evacuated. The pleural fluid did not contain food particles, pH was 7, and amylase was 685 U/L. Gastroscopy confirmed an oesophageal tear in the lower one-third of the oesophagus. At that time, a severe concomitant mucosal inflammation precluded further endoscopic treatment because of a too high risk of oesophageal rupture. A control CT scan demonstrated a left-sided paravertebral encapsulated air-fluid collection and a left pleural effusion that had substantially increased in size. A pigtail catheter was inserted into the left pleural space. CT guided drainage of encapsulated pleural collections was performed. Four days after ICU admission, a self-expandable oesophageal stent was endoscopically placed. Despite stenting, continuous bilateral pleural drainage, and broad antimicrobial covering, the patient remained septic with hectic fever and persistently high leukocyte counts and CRP levels. Video-assisted thoracoscopic surgery was performed with drainage and rinsing of the mediastinum and pleural cavities. Thereafter, fever subsided and clinical and biological parameters progressively normalised. After an ICU stay of 33 days, the patient was discharged.

## 3. Discussion

Boerhaave's syndrome is a barogenic tear of the oesophagus caused by a sudden rise of intraluminal pressure in its distal end. It accounts for approximately 15% of all cases of oesophageal rupture and has a mortality rate up to 40%. In the majority of cases, the rupture is located in the left posterolateral wall of the distal third of the oesophagus [2].

Boerhaave's syndrome is readily suspected in a patient with a history of overindulgence in food or drinks who, after severe or repeated vomiting, experiences excruciating chest pain and develops subcutaneous emphysema [3]. However, up to one-third of patients have atypical symptoms or are admitted with severe respiratory distress and/or shock. The differential diagnosis of Boerhaave's syndrome includes a variety of acute thoracic and abdominal conditions including myocardial infarction, pulmonary embolus, dissecting aorta, ruptured aortic aneurysm, perforated peptic ulcer, Mallory-Weiss syndrome, pancreatitis, pneumonia, and spontaneous pneumothorax [4].

On physical examination, patients generally appear acutely ill with tachycardia and tachypnea. Fever may be present or not. Auscultation occasionally reveals decreased breath sounds on the perforation side. When mediastinal emphysema is present, Hamman's sign (i.e., a mediastinal “crackling” accompanying every heart beat) may be heard in left lateral decubitus position [2]. 

Blood laboratory tests are of  little help to support the diagnosis, except for excluding other pathologies (e.g., myocardial infarction, pancreatitis). Presence of food particles, a high amylase content, and a low pH in pleural fluid are either highly confirmatory or very suggestive of oesophageal perforation [2, 3]. 

Imaging is of key importance for diagnosis. Plain chest X-rays may reveal subcutaneous and/or mediastinal emphysema, mediastinal widening, pleural effusion(s), pneumothorax, hydrothorax, and intrathoracic air-fluid levels or masses [5]. In 20% of cases, a “V sign” is noticed which appears as a radiolucent streak of air dissecting the retrocardiac fascial planes [6]. A rapidly developing or evolving effusion requires urgent investigation. Importantly, up to 15% of patients have normal chest roentgenograms. CT scanning of thorax and mediastinum has largely replaced the former gastrografin swallow tests. CT indeed allows a more detailed assessment of the lungs, mediastinum, pleura, and aorta. CT also is more sensitive than plain radiography for detection of small amounts of paraspinal or pleural air-fluid collections [7]. Finally, endoscopy enables direct visualisation of the location and extent of the perforation but must be performed with caution since it may potentially aggravate the oesophageal tear.

**Figure 4:**
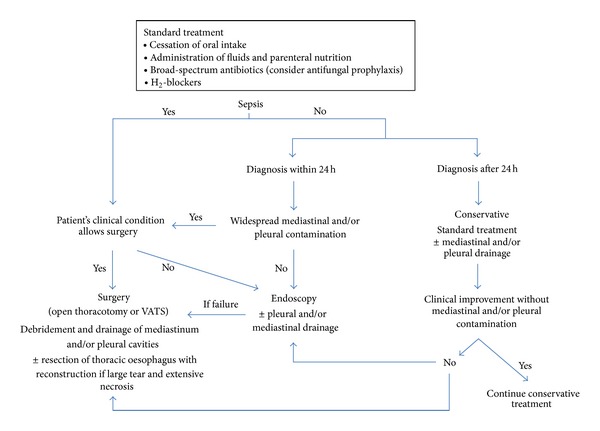
Boerhaave's syndrome treatment algorithm.

Management of Boerhaave's syndrome remains a controversial issue. Basically, three levels of treatment are distinguished: a conservative, an endoscopic, and a surgical approach [8, 9]. Conservative treatment consists of cessation of oral intake, administration of fluids and parenteral nutrition, broad-spectrum antibiotics, H_2_-blockers, and eventual mediastinal, pleural, or abscess drainage. It can be offered to selected, nonseptic patients with a small or well-contained perforation [10]. Endoscopic therapy is increasingly used in patients whose perforation is diagnosed early without widespread contamination and sepsis. Endoluminal placement of a self-expandable metallic stent to bridge an oesophageal tear has shown encouraging results. Yet, endoscopic stenting in Boerhaave's syndrome has a rather undetermined and center-dependent success rate and may not be devoid of side effects such as enhanced mediastinal or pleural contamination and accidental stent migration [11]. Finally, surgical treatment ranges from a less invasive approach consisting of debridement and drainage of mediastinum and pleural cavities to extensive resection of the thoracic oesophagus [8, 9, 12]. Factors determining surgical intervention are the extent of the perforation, eventual concomitant pathologies that require synchronous management, the degree of mediastinal or pleural contamination, and the presence of sepsis. Abbas et al. advocate primary surgical repair when patients present with sepsis, large uncontained leaks, and extensive contamination [13]. Based on a literature review, de Schipper et al. propose to perform surgery (i.e., open thoracotomy with resection, hemifundoplication, and pleural/mediastinal drainage) in patients diagnosed early (<48 h), regardless sepsis is present or not, and in patients diagnosed beyond 48 h who remain or become septic under conservative treatment [14]. Experience with less invasive surgical procedures is scarce. Haveman et al. compared open thoracotomy with video-assisted thoracoscopic surgery (VATS) and found similar success rates [15]. More prospective studies are warranted to establish the potential advantage of VATS in treating patients with Boerhaave's syndrome. To deal with single incident cases, a treatment algorithm is composed and presented in Figure 4. According to this algorithm, it could be argued that early surgical intervention might have been the preferred optimal first-line treatment in our patient.

## 4. Conclusion

Boerhaave's syndrome still represents a diagnostic and therapeutic challenge. Timely recognition shouldered by repeated radiological imaging is an important prognostic determinant. The choice of treatment depends upon critical and continuous assessment of the patient's clinical status, the extent and duration of the leak, and the presence of sepsis. Minimally or less invasive treatment options have been applied successfully, but their impact must be evaluated in prospective trials.
